# Clinical study on wound healing properties of Nile tilapia fish skin as biological dressing in dogs

**DOI:** 10.1371/journal.pone.0286864

**Published:** 2025-02-28

**Authors:** Khizer Ahmed Khan, Uzma Farid Durrani, Asim Khalid Mahmood, Muhammad Yasin Tipu, Amber Fatima, Hussain Ahmad Saeed, Abdul Karim Khalid, Tuba Shuja Ansari

**Affiliations:** 1 Department of Small Animal and Clinical Sciences, University of Veterinary and Animal Sciences, Lahore, Pakistan; 2 Department of Pathology, University of Veterinary and Animal Sciences, Lahore, Pakistan; Universidade de Trás-os-Montes e Alto Douro: Universidade de Tras-os-Montes e Alto Douro, PORTUGAL

## Abstract

Frequency of clinical cases of dogs with massive skin losses is very high in urban areas of Pakistan following road accidents, sharp objects exposure and attack by other dog. These cases need intensive veterinary assistance for safe and speedy healing of wounds. Recently, skin of Nile tilapia fish *(Oreochromis niloticus)* is internationally gaining hype in medical field as biological dressing to boost dermatological reconstruction process. Nile tilapia skin is a recent research trend and a very limited research data is available on this topic for both human and animal subjects. This study was conducted at Department of Small Animal Clinical Sciences, University of Veterinary and Animal Sciences (UVAS), Lahore, Pakistan considering the wound healing promoter properties of Nile tilapia skin as a biological dressing for dogs with massive skin losses. Aim of this study was to evaluate Nile tilapia fish skin as wound healing promoter biological dressing following sutured and non-sutured application techniques. For this study 10 clinical cases of dogs were randomly selected as per set criteria and divided into groups A and B comprising 5 dogs each. Consent document was signed by each dog owner for volunteer participation in this study. Nile tilapia skin was collected from fresh subjects and treated with 10% povidone-iodine for 10-15 minutes to prepare biological dressing. In group A, biological dressing was sutured on wound (non-absorbable silk suture material) on the area with dermal loss. In group B, biological dressing was applied in a wrap manner on area of dermal loss without application of sutures. Wound healing was evaluated grossly and histologically on days 0, 7 and 14. Statistical analysis of comparison between groups A and B revealed that application of Nile tilapia skin derived biological dressing in wrap fashion results in fast and complication-free wound healing as compared to sutured tilapia biological dressing in dogs.

## Introduction

Nile tilapia (*Oreochromis niloticus*) is a fresh water fish originated from the Nile basin. Tilapia is known as a disease resistant; most cultured fish worldwide (East Africa). Nile tilapia skin is a rich source of gelatin and collagen that make it a valuable biomaterial for tissue restoration in dogs. Biological dressings like xenografts, allografts, cultured wound healing cells, i.e., allogenic cultured epidermis (allo-CE) and bio-engineered tissues already being practiced for topical treatment of skin burns and wounds [1]. Current trend shows that soon collagen rich dressings will replace the traditional biological dressings due to lower antigenicity, frequent hemostasis, accelerated fibroplasia and rapid epithelialization. Tilapia skin is easily available and in-expensive biological material that possesses bio-compatible type 1 collagen which has potential for use in clinical regenerative procedures [2].

Nile tilapia skin is an effective option for the management of extensive wounds due to its non-infectious microbiota, structural similarity to human skin and clinical success is reported when this is used as a biological dressing for the treatment of burns and excessive dermatological losses [3]. It is hypothesized that similar result can be achieved with the use of Nile tilapia skin as biological dressing in dogs for restoration of dermatological losses [4]. reported that tilapia skin grafts can be successfully used for management of large wounds in dogs and may promote accelerated epithelialization in full thickness skin wounds.

Application of Nile tilapia skin derived biological dressing on extensive wounds provides safe and effective skin replacement, free of viral and prion disease transmission risk [5]. Tilapia skin resembles mammalian skin due to its maintained protein structure. This is extremely porous that provides an extracellular matrix composed of glycosaminoglycans, proteoglycans, fibronectins and growth factors that manage migration of autologous cells to promote proliferative and epithelialization phase of the healing process. 

Tilapia skin is also rich in Omega-3 polyunsaturated fatty acids (PUFAs) [6]. reported that eicosapentaenoic acid (EPA) and docosahexaenoic acid (DHA) act as effective antimicrobial agent and also regulate inflammatory response of crucial stage of acute wound healing [7]. These fatty acids partly inhibit a number of aspects of inflammation including leukocyte chemotaxis, adhesion molecule expression and leukocyte–endothelial adhesive interactions, production of prostaglandins and leukotrienes from the n −  6 fatty acid arachidonic acid, production of inflammatory cytokines and T-helper 1 lymphocyte reactivity [8].

Wound healing is a challenging and continuous process that consists of four phases, i.e., coagulation and hemostasis, inflammation, proliferation, wound remodeling followed by formation of scar tissue. Once Nile tilapia skin is applied, it starts accelerating wound healing by decreasing the effects of pro-inflammatory mediators like tumor necrotic factor-α (TNFα), Interleukin-6 (IL-6), and Interleukin-8 (IL-8) and increases the number of receptors on target cells for β-defense 14, pattern recognition receptor (NOD2), anti-inflammatory Interleukin-10 (IL-10), vascular endothelial growth factor and fibroblast growth factor (β-FGF) [9]. Nile tilapia skin also increases the bacterial growth responsible to promote wound healing and decreases bacterial growth that disturbs wound healing [7].

Nile tilapia fish skin needs less processing steps to prepare a ready to made biological dressing. It results in preservation of three-dimensional structure and reflect a normal, non-infectious colonization/microbiota, i.e., microbial load less than 100,000 CFU/g of tissue; a cut off standardized value for the American Society of Microbiology to differentiate the causative agent from the colonizers [10].

There is a lack of clinical data on tilapia skin as wound healing promoter biological dressing in small animals. The present study was conducted to evaluate and compare the effectiveness of sutured and non-sutured Nile tilapia skin derived biological dressing as dermatological wound healing promoter in dogs.

## Materials and methods

### Ethical approval

The study was approved by Ethical Review Committee, University of Veterinary and Animal Sciences (UVAS), Lahore, Pakistan following a thorough review and confirmation that all the procedures in this study are in accordance with the Pakistani by laws and OIE animal welfare standards for animal care and use in education and research. The written consent of the participants was acquired on consent document for record purpose.

### Study animals

The study was conducted on 10 randomly selected clinical cases of vaccinated dogs received at Department of Small Animal Clinical Sciences, University of Veterinary and Animal Sciences (UVAS), Lahore, Pakistan with different types of wounds. Consent from owners of participating dogs was acquired on prescribed document and dogs were housed in standard housing facilities at department kennels. Dogs selected for this study were randomly divided into 2 groups, i.e., A & B, each group with 5 dogs. Group A was treated with sutured Nile tilapia skin as biological dressing while group B was treated with non-sutured Nile tilapia skin.

### Collection & processing of Nile tilapia fish skin

Fish skin was collected from fresh Nile tilapia (*Oreochromis niloticus*) (weighing: approx. 300-650 grams), acquired from Tawakkal Tilapia Hatchery, Muzaffargarh, Pakistan. Fish was euthanized following pithing and decapitation and transported in ice sealed container to UVAS, Lahore, Pakistan. In processing room fish scales were removed by knife and blunt scrapping of fish was performed. Fish skin was dissected using scalpel blade no. 24 and released from the underlying structures (muscles, tissues.). Skin was incised into strips of size and shape of recipient site and placed in normal saline solution to remove any impurities. Collected skin strips were subjected to sterilization by 10% povidone-iodine dipping for 10-15 minutes contact time followed by placing in sterile normal saline to make ready to be applied [1].

### Preparation of recipient site

Before application of tilapia skin each dog was generally anesthetized, i.e., induction with injection Propofol (200mg/20mL) at dose rate of 6-7 mg/kg intravenous (cephalic vein) followed by anesthesia maintenance with Isoflurane (inhalation) at dose rate of 1.5% - 1.8%. Recipient site was cleaned with Luke warm sterile normal saline. Necrotic debris and other contaminations were removed. Surgical area and surgical site was prepared before procedure. Ketoprofen at dose rate of 2-3mg/kg IM, was injected for pain management in dogs (days 1, 2, 3 post-procedure and on days of bandage change).

### Application of Nile tilapia skin

In Group A, under controlled environment of operation room; sterilized fish skin was placed on wound and sutured with non-absorbable suture, i.e., silk (size 3-0) by using simple interrupted suture pattern and covered with traditional bandage. Elizabethan collar was used in each dog to avoid scratch or bites on biological dressing. While in Group B, sterilized fish skin was placed on wound and applied by simple wrapping followed by bandage application. Signs of individuals body reaction to biological dressing of Nile tilapia fish skin may include; skin erythema, edema, swelling, and itching.

### Sample collection by punch biopsy

**Fig 1:**
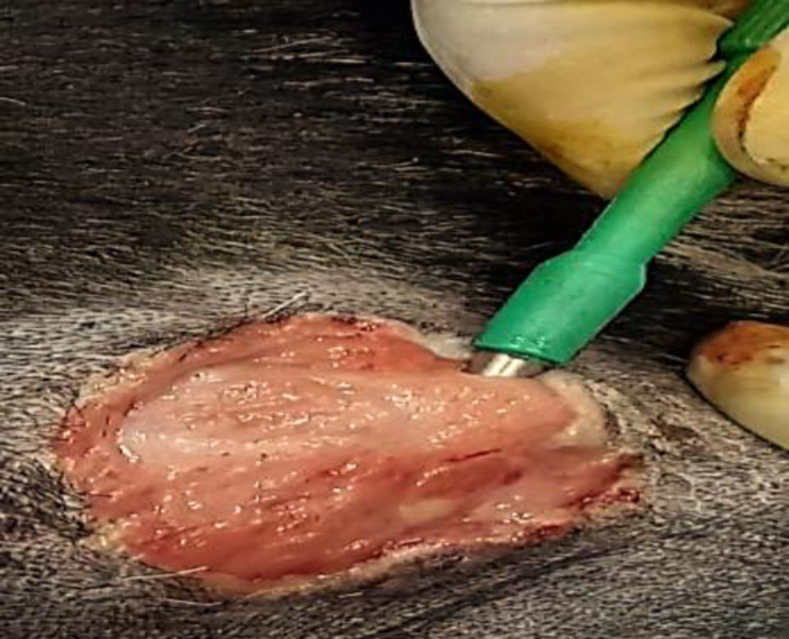
Punch biopsy for histopathological findings of wound in dog.

Biopsy punch (6mm) for each specimen was used as per decided schedule (Table 1). Punch biopsy was performed in the center of wound by uni-directional rotation. Biopsy punch was removed & gently tissue section was grasped with the tissue forceps and placed in a labeled sample container having 10% neutral buffered formalin [11]. Specimens were shifted to histopathology laboratory for tissue processing & microscopic evaluation in the Department of Pathology, UVAS, Lahore, Pakistan (Fig 1).

**Table 1:** Post-procedure follow-up protocol.

Groups	General evaluation days	Days of wound measurement	Histopathology schedule (Punch biopsy)
A	Daily	0, 7 & 14	0, 7 & 14
B	Daily	0, 7 & 14	0, 7 & 14

### Parameters of study

**Fig 2:**
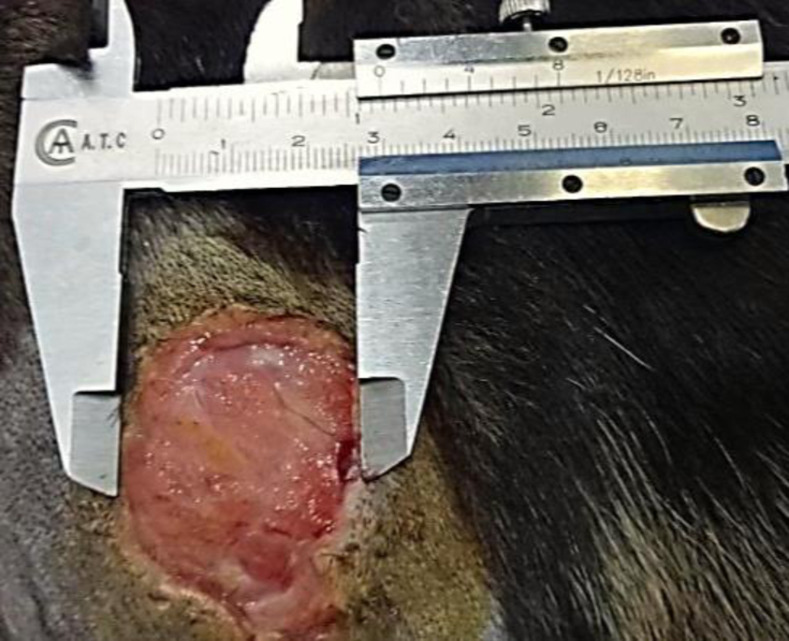
Wound measurement in dog by vernier caliper.

It included pre and post-treatment wound size measurement (Fig 2), percentage of wound contraction =  100 – total wound area/original wound area x 100 [12] and quality of wound healing (healing speed, scar formation, sterility of wound) was determined by using the percentage of wound contraction on days 0, 7 and 14.

### Statistical analysis

Data was analyzed statistically using General Linear Model of SAS for statistical differences (p <  0.05). Post hoc analysis was done by Duncan Multiple Range (DMR) test.

## Results

Present study was carried out to evaluate the effectiveness of Nile tilapia skin as biological dressing to promote healing of various dermatological losses in dogs.

### Group A: Sutured biological dressing

Wound healing of dogs treated with sutured Nile tilapia fish skin as biological dressing was evaluated as per following parameters

#### Wound size.

Wound size was measured in each dog on days 0, 7 & 14. Mean wound size on days 0, 7 & 14 was 18.38 ±  4.90, 15.44 ±  4.34, 12.80 ±  4.27 respectively. Statistical analysis showed a significant difference (P <  0.05) among mean size of wound healing area from day 0 -14.

#### Physical characteristics of wound.

The members in this group were presented with different wound appearances in terms of shape, size and healing status (Table 2). In all cases, wounds were not more than 24 hours old as [13] Conducted a study and stated that there is polymorphonuclear leukocyte activity in chronic wounds which may impair wound healing process.

**Table 2:** Measurements and Physical characteristics of wounds.

No. of Dogs	Mean Wound Size Area (cm)	Shape of Wound	Status Infected/ Non Infected
Dog 1	9.45	Elliptical	Non infected
Dog 2	10.50	Irregular	Non infected
Dog 3	36.45	Irregular	Non infected
Dog 4	15.37	Elliptical	Non infected
Dog 5	20.13	Circular	Non infected

#### Quality of wound healing.

**Fig 3:**
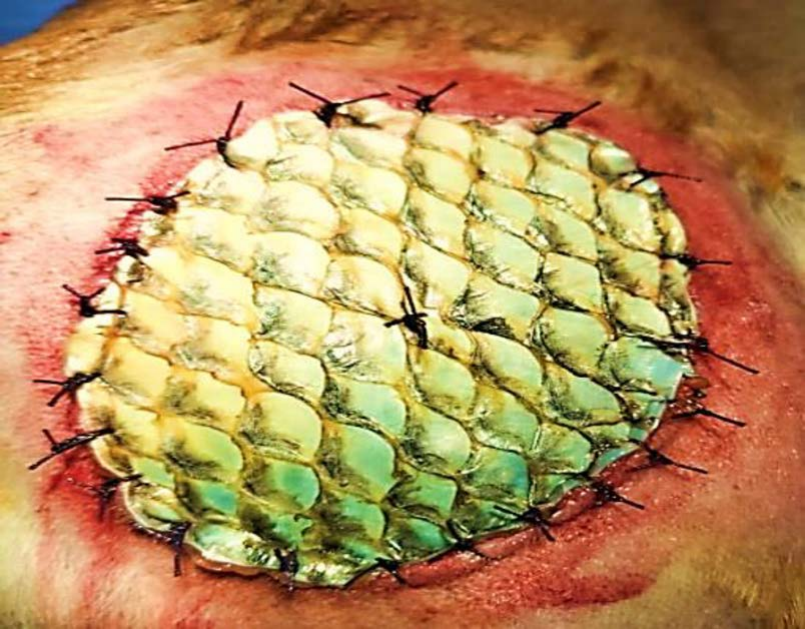
Biological dressing of sutured Nile tilapia fish skin on right thigh region of dog (8.1× 4.5 cm).

Out of total 5 dogs, only 1 dog showed mild scar formation. All other 4 dogs showed no scar formation. Bandage was observed twice a day and changed on alternate days. Tilapia skin biological dressing was cleaned with normal saline in order to keep biological dressing and wound clean to avoid contamination and risk of infection. During the whole course of treatment, no dog showed any sign of reaction to Nile tilapia biological dressing, e.g., skin erythema, edema, swelling, and itching. According to the statistical analysis, the relation between mean wound size of this group with respect to days has shown a significant difference that indicates fast wound healing process (Fig 3).

#### Histopathological findings.

**Fig 4:**
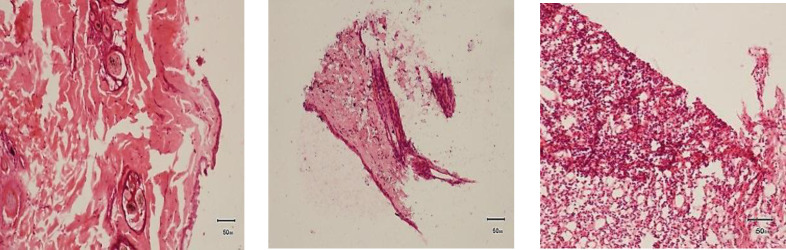
Pre and post treatment histopathological evaluation of wounds at different intervals. (A) Complete absence of epidermis (B) Epidermal regeneration seen (C) Intact epidermis (1) with small intraepidermal vesicle is seen (2), keratohyaline granules are large and numerous (3) and mature keratin is visible (4).

Histopathological changes showed epithelialization were in progress (Fig 4A-4C).

### Group B: Non Sutured Biological Dressing

#### Wound size.

Wound size was measured in each dog on days 0, 7 and 14. Mean wound size on days 0, 7 & 14 was 17.93 ±  5.48, 11.48 ±  3.95, 6.98 ±  2.59, respectively. Statistical analysis showed a significant difference (P <  0.05) among mean size of wound healing areas from day 0-14.

#### Physical characteristics of wound.

**Fig 5:**
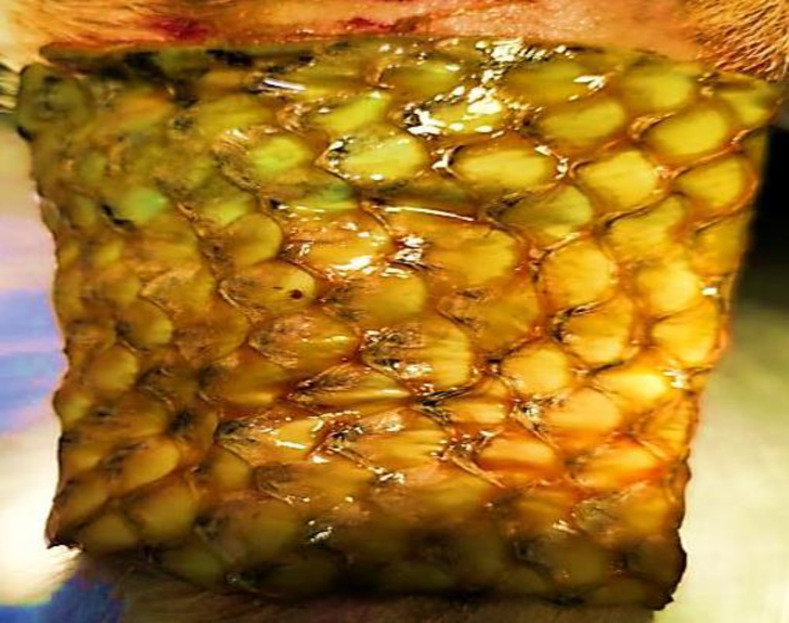
Biological dressing of non-sutured Nile tilapia fish skin in dog skin on left forelimb of dog (6.7×  3.8 cm).

The members in this group were presented with different wound appearances in terms of shape, size and healing status (Table 3). All 5 cases were accidental injuries with wounds not more than 24 hours old (Fig 5).

**Table 3:** Measurements and Physical characteristics of wounds.

No. of Dogs	Mean Wound Size Area (cm)	Shape of Wound	Status Infected/ Non Infected
Dog 1	3.42	Oval	Non infected
Dog 2	5.70	Elliptical	Non infected
Dog 3	26.98	Irregular	Non infected
Dog 4	25.46	Elliptical	Non infected
Dog 5	28.08	Circular	Non infected

#### Quality of wound healing.

Out of total 5 dogs, no dog showed scar formation. Bandage was changed on alternate days. During changing of bandage, normal saline was poured on biological dressing of Nile tilapia skin in order to prevent it from peeling and to minimize any risk of bacterial infection. During the whole course of treatment, no dog showed any sign of reaction to Nile tilapia biological dressing, e.g., skin rashes, swelling, and itching.

#### Histopathological findings.

**Fig 6:**
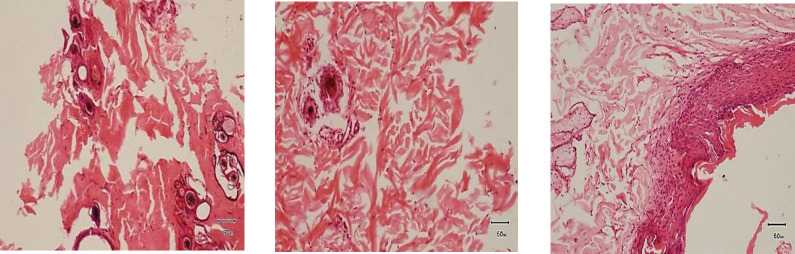
Pre and post treatment histopathological evaluation of wounds at different intervals. (A) Complete absence of epidermis. (B) Active cells of dividing stratum basale and layers of stratum spinosum had formed. (C) Rapid epithelialization with multiple layers of growing epithelium (1) along with granulation tissue is seen (2).

Histopathological changes showed epithelialization were in progress (Fig 6A-6C).

## Mean wound size comparison between group A and B

**Fig 7:**
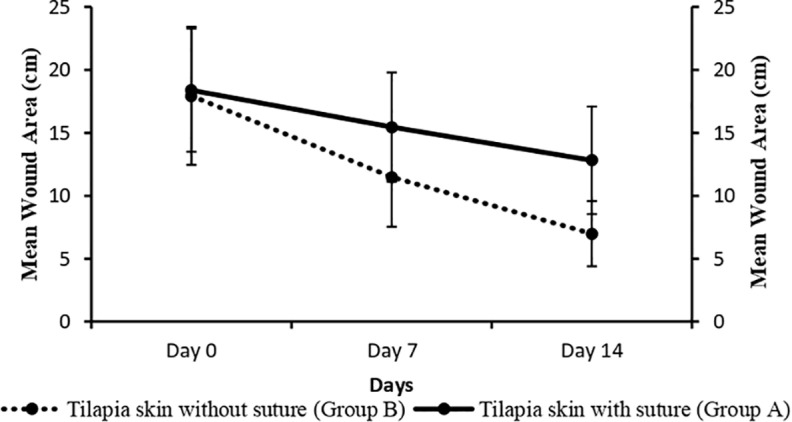
Mean wound size comparison between Group A and B.

Mean wound size was measured on days 0, 7 and 14 in group A and B. Statistical data showed significant difference (P <  0.05) among wound size from days 0 - 14 within each group but data was non-significant (P >  0.05) between groups A and B. In group A, mean wound size on day 0, 7 and 14 was 18.38 ±  10.96, 15.44 ±  10.96 and 12.80 ±  9.55 respectively. In group B, mean wound size on day 0, 7 and 14 was 17.93 ±  12.26, 11.48 ±  8.83 and 6.98 ±  5.79 respectively (Fig 7). In graphical presentation, the details of the mean wound size indicated that group A had difference with group B which means more rapid healing of group B than group A.

## Discussion

Wound healing process does not elicit pain and tilapia skin induces anti-nociceptive properties in recently conducted studies [14]. Current study revealed that tilapia fish skin applied as biological dressing on clinical cases of dogs with various degrees of dermatological losses accelerated wound epithelialization resulted in rapid wound healing. Also, it inhibited exuberant granulation tissue formation, wound contamination, tilapia skin biological dressing sensitivity reaction, and minimized frequent dressing changes [15].

Sterilization of biological dressing of Nile tilapia skin is important prior to its application on recipient wound [1]. conducted a study and reported that the use of 2% chlorhexidine and 10% povidone-iodine is equally effective and a study conducted by [16] reported that sample immersion in 2% chlorhexidine did not cause collagen alterations on tilapia skin.

[3] Conducted a study by using tilapia skin as a xenograft for partial thickness burns after a gunpowder explosion in human patient and stated it takes 12 to 17 days for complete re-epithelialization of patients wounds in upper limbs and no dressing changes due to good adherence of tilapia skin to the wound bed. So, similar strategy was adapted and present study was conducted for 14 days. Fish skin dressings were changed twice per week in summer clinical trials and once per week in winter clinical trials. A study conducted by [3] Reported that fish skin dressings did not need to change regularly as the conventional dressing, also, it can stay up to 10 days. Reduction in time of wound dressing may lessen stress and pain associated with change in dressing every time especially in cases of chronic wounds. Once bleeding stops, newly formed clot and surrounding tissue releases pro-inflammatory cytokines and growth factors within 24 hours. Inflammatory cells migrate to the wounds to promote inflammatory phase of healing. Neutrophils infiltrate the wound within one hour and sustain level for the first 48 hours. Macrophages are phagocytic cells that reach highest concentration in wound within 48–72 hours following the injury whereas Lymphocytes appear after 72 hours [17].

Biological dressing of Nile tilapia skin kept the wounds hydrated and moist as an occlusive dressing to maintain tissue humidity, which is needed for rapid epithelialization [18]. Present study showed that tilapia skin dressing allowed influx of the epithelial cells, resulted in reduction of wound size and epithelial gap between wound margins. Tilapia skin dressing is rich in collagen and amino acids, such as alanine and silk that enhances formation of granulation tissue, proliferation of fibroblasts and collagen coalescence in the wound.

Present study revealed that the biological dressing of Nile tilapia fish skin fastened wound healing process to repair various dermatological losses in dogs as [19] conducted a study on use of Nile tilapia skin as dermal wound healing promoter in cats and concluded sutured biological dressing of Nile tilapia fish skin proved to be highly effective for safe, rapid and aseptic wound healing as compared to open wound healing process for dermal losses in cats. More studies are needed to further investigate the utility of this technique.

## Conclusion

Use of Nile tilapia skin as biological wound dressing is an effective and reliable technique to promote repair and healing of dermatological damage/losses in dog. Non-sutured biological dressing of Nile tilapia skin shows rapid healing response without formation of exuberant granulation tissue. This is found to be more effective for fast, safe and infection free healing of the wound as compared to the sutured biological dressing of Nile tilapia fish skin.

In future additional studies are needed to investigate tilapia skin healing properties in veterinary clinical field that will also support the human medical practice.
